# Temporal Trends in Acute Adrenal Insufficiency Events in Children With Congenital Adrenal Hyperplasia During 2019-2022

**DOI:** 10.1210/jendso/bvae145

**Published:** 2024-08-20

**Authors:** Xanthippi Tseretopoulou, Salma R Ali, Jillian Bryce, Nadia Amin, Navoda Atapattu, Tania A S S Bachega, Federico Baronio, Rita Ortolano, Niels H Birkebaek, Walter Bonfig, Martine Cools, Justin H Davies, Tessy Thomas, Liat de Vries, Heba Elsedfy, Nermine H Amr, Christa E Flueck, Evgenia Globa, Tulay Guran, Zehra Yavas-Abali, Ayla Guven, Sabine E Hannema, Violeta Iotova, Daniel Konrad, Nina Lenherr-Taube, Nils P Krone, Sofia Leka-Emiri, Elpis Vlachopapadopoulou, Corina Lichiardopol, Otilia Marginean, Renata Markosyan, Uta Neumann, Marek Niedziela, Magdalena Banaszak-Ziemska, Franziska Phan-Hug, Sukran Poyrazoglu, Ursina Probst-Scheidegger, Tabitha Randell, Gianni Russo, Mariacarolina Salerno, Sumudu Seneviratne, Margarett Shnorhavorian, Ajay Thankamony, Rieko Tadokoro-Curraro, Erica van den Akker, Judith van Eck, Ana Vieites, Malgorzata Wasniewska, S Faisal Ahmed

**Affiliations:** Developmental Endocrinology Research Group, School of Medicine, Dentistry & Nursing, University of Glasgow, Glasgow G51 4TF, UK; Office for Rare Conditions, Royal Hospital for Children & Queen Elizabeth University Hospital, Glasgow G51 4TF, UK; Developmental Endocrinology Research Group, School of Medicine, Dentistry & Nursing, University of Glasgow, Glasgow G51 4TF, UK; Office for Rare Conditions, Royal Hospital for Children & Queen Elizabeth University Hospital, Glasgow G51 4TF, UK; Office for Rare Conditions, Royal Hospital for Children & Queen Elizabeth University Hospital, Glasgow G51 4TF, UK; Department of Paediatric Endocrinology, Leeds Teaching Hospitals NHS Trust, Leeds LS1 3EX, UK; Department of Paediatric Endocrinology, Lady Ridgeway Hospital, Colombo 00800, Sri Lanka; Unidade de Endocrinologia do Desenvolvimento, Laboratório de Hormônios e Genética Molecular/LIM42, Disciplina de Endocrinologia, Hospital Das Clinicas, Faculdade De Medicina, Universidade de Sao Paulo, São Paulo, 05508-090, Brazil; Department Hospital of Woman and Child, Pediatric Unit, IRCCS—Azienda Ospedaliero-Universitaria di Bologna, 40138 Bologna, Italy; Department Hospital of Woman and Child, Pediatric Unit, IRCCS—Azienda Ospedaliero-Universitaria di Bologna, 40138 Bologna, Italy; Department of Paediatrics, Aarhus University Hospital, Aarhus DK-8200, Denmark; Department of Paediatrics, Technical University München, D-80804 Munich, Germany; Department of Paediatrics, Klinikum Wels-Grieskirchen, A-4600 Wels, Austria; Department of Paediatric Endocrinology, University Hospital Ghent, Ghent University, 9000 Ghent, Belgium; Department of Paediatric Endocrinology, University Hospital Southampton, Southampton SO16 6YD, UK; Department of Paediatric Endocrinology, University Hospital Southampton, Southampton SO16 6YD, UK; The Jesse and Sara Lea Shafer Institute of Endocrinology and Diabetes, Schneider Children's Medical Center of Israel, Petah Tikvah 4920235, Israel; Faculty of Medical&Health Sciences, Tel Aviv University, Tel Aviv 6997801, Israel; Department of Pediatrics, Ain Shams University, 11566 Cairo, Egypt; Department of Pediatrics, Ain Shams University, 11566 Cairo, Egypt; Pediatric Endocrinology, Diabetology and Metabolism, Department of Pediatrics, Bern University Hospital Inselspital, University of Bern, 3010 Bern, Switzerland; Pediatric Endocrinology, Diabetology and Metabolism, Department of BioMedical Research, Bern University Hospital Inselspital, University of Bern, 3010 Bern, Switzerland; Ukrainian Scientific and Practical Center of Endocrine Surgery, Transplantation of Endocrine Organs and Tissues of MOH of Ukraine, Kyiv 01021, Ukraine; Department of Pediatric Endocrinology and Diabetes, Marmara University, 34899 Pendik, Istanbul, Turkey; Department of Pediatric Endocrinology and Diabetes, Marmara University, 34899 Pendik, Istanbul, Turkey; Department of Paediatric Endocrinology, Baskent University Medical Faculty, Istanbul Hospital, 06790 Istanbul, Turkey; Department of Pediatric Endocrinology, Amsterdam UMC location Vrije Universiteit Amsterdam, 1007 MB Amsterdam, The Netherlands; Department of Paediatrics, Medical University-Varna, UMHAT “Sv. Marina,” 9002 Varna, Bulgaria; Department of Endocrinology and Diabetology, University Children's Hospital Zurich, University of Zurich, 8032 Zurich, Switzerland; Department of Endocrinology and Diabetology, University Children's Hospital Zurich, University of Zurich, 8032 Zurich, Switzerland; Department of Oncology and Metabolism, University of Sheffield, Sheffield S10 2RX, UK; Department of Endocrinology-Growth and Development, “P&A Kyriakou” Children's Hospital, Athens 115 27, Greece; Department of Endocrinology-Growth and Development, “P&A Kyriakou” Children's Hospital, Athens 115 27, Greece; Department of Endocrinology, University of Medicine and Pharmacy Craiova, University Emergency Hospital, Craiova 200349, Romania; Department of Paediatrics, University of medicine and Pharmacy “Victor Babes”, Clinical children emergency hospital “Louis Turcanu” Timisora, Timisora 300011, Romania; Department of Endocrinology, YSMU, Yerevan 0025, Armenia; Department of Paediatric Endocrinology and Diabetology, Centre for Chronic Sick Children, Institute for Experimental Paediatric Endocrinology, Charité-Universitätsmedizin Berlin, 10117 Berlin, Germany; Department of Pediatric Endocrinology and Rheumatology, Institute of Pediatrics, Poznan University of Medical Sciences, 61 701 Poznan, Poland; Department of Pediatric Endocrinology and Rheumatology, Institute of Pediatrics, Poznan University of Medical Sciences, 61 701 Poznan, Poland; Paediatric Endocrinology, EHC-Morges, 1110 Morges, Switzerland; Paediatric Endocrinology, Hospital Valais-Sion, 1950 Sion, Switzerland; Istanbul Faculty of Medicine, Department of Paediatrics, Paediatric Endocrinology Unit, Istanbul University, 34093 Çapa, Istanbul, Turkey; Department of Paediatric Endocrinology, Kantonsspital Winterthur, 8401 Zurich, Switzerland; Department of Paediatric Endocrinology, Nottingham Children's Hospital, Nottingham NG7 2UH, UK; Department of Paediatrics, Endocrine Unit, RCCS Ospedale San Raffaele, 20132 Milan, Italy; Department of Pediatrics, Unit of Immunology, Federico II University, 80131 Naples, Italy; Department of Paediatrics, Faculty of Medicine, University of Colombo, Colombo 00700, Sri Lanka; Department of Urology, University of Washington School of Medicine, Seattle Children's Hospital, Seattle, WA 98105, USA; Department of Paediatrics,University of Cambridge, Cambridge CB2 2QQ, UK; Department of Paediatrics,University of Cambridge, Cambridge CB2 2QQ, UK; Division of Pediatric Endocrinology, Department of Pediatrics, Erasmus MC-Sophia Children's Hospital, 3015 CN Rotterdam, The Netherlands; Division of Pediatric Endocrinology, Department of Pediatrics, Erasmus MC-Sophia Children's Hospital, 3015 CN Rotterdam, The Netherlands; Centro de Investigaciones Endocrinológicas, División de Endocrinología, Hospital de Niños Ricardo Gutiérrez, 1425 Buenos Aires, Argentina; Pediatric Unit, Department of Human Pathology of Adulthood and Childhood, University of Messina, 98166 Messina, Italy; Developmental Endocrinology Research Group, School of Medicine, Dentistry & Nursing, University of Glasgow, Glasgow G51 4TF, UK; Office for Rare Conditions, Royal Hospital for Children & Queen Elizabeth University Hospital, Glasgow G51 4TF, UK

**Keywords:** 21-hydroxylase deficiency, adrenal insufficiency, adverse events, benchmark, congenital adrenal hyperplasia, sick day episodes, quality improvement, registry

## Abstract

**Background:**

It is unclear whether targeted monitoring of acute adrenal insufficiency (AI) related adverse events (AE) such as sick day episodes (SDEs) and hospitalization rate in congenital adrenal hyperplasia (CAH) is associated with a change in the occurrence of these events.

**Aim:**

Study temporal trends of AI related AE in the I-CAH Registry.

**Methods:**

In 2022, data on the occurrence of AI-related AE in children aged <18 years with 21-hydroxylase deficiency CAH were compared to data collected in 2019.

**Results:**

In 2022, a total of 513 children from 38 centers in 21 countries with a median of 8 children (range 1-58) per center had 2470 visits evaluated over a 3-year period (2019-2022). The median SDE per patient year in 2022 was 0 (0-2.5) compared to 0.3 (0-6) in 2019 (*P* = .01). Despite adjustment for age, CAH phenotype and duration of study period, a difference in SDE rate was still apparent between the 2 cohorts. Of the 38 centers in the 2022 cohort, 21 had also participated in 2019 and a reduction in SDE rate was noted in 13 (62%), an increase was noted in 3 (14%), and in 5 (24%) the rate remained the same. Of the 474 SDEs reported in the 2022 cohort, 103 (22%) led to hospitalization compared to 299 of 1099 SDEs (27%) in the 2019 cohort (*P* = .02).

**Conclusion:**

The I-CAH Registry can be used for targeted monitoring of important clinical benchmarks in CAH. However, changes in reported benchmarks need careful interpretation and longer-term monitoring.

Congenital adrenal hyperplasia (CAH) resulting from 21-hydroxylase deficiency (21-OHD) is the most common genetic cause of adrenal insufficiency (AI) in childhood with a reported worldwide incidence ranging between 1 in 14 000 and 1 in 18 000 births [1]. Acute AI-related adverse events (AEs), including sick day episodes (SDEs) and adrenal crises (AC) are a major cause of morbidity in these patients with a reported incidence of 2.7 and 10.9 per 100 person-years [2-6]. Reduction of acute AI-related AEs may be achieved through optimization of therapy and prevention of acute sequelae with new therapies often aiming to have a beneficial impact on acute AI-related AEs [7, 8]. The cornerstone of prevention of acute events also includes structured patient and parental education, family support events, and provision of a steroid emergency plan and cards [9, 10]. Temporal trends of adrenal insufficiency have been studied but have mainly focused on hospital admissions and adrenal crises in adolescents and young adults [11, 12]. Over the past few years, the International CAH Registry (I-CAH) has increasingly demonstrated its ability to use routinely collected real-world data to create clinical benchmarks and understand geographical and temporal trends in routine clinical practice and core clinical outcomes such as acute AE-related AI events [6, 13-15]. Centers that participate in this registry employ a range of measures that may improve the quality of care [6]. Following an initial study of the occurrence of AI-related AEs that led to a creation of a benchmark of these events among participating centers, individual centers were provided with a center-specific benchmarking report with information on the quality of care, as reflected by SDEs, adrenal crises, and hospitalization rates [16]. The aim of the current study was to investigate temporal changes in the occurrence of acute AI-related AEs since the initial benchmarking exercise.

## Methods

### Study Population

All patients younger than age 18 years with visits during a 3-year period from August 2019 who were registered as having 21-OHD CAH were identified from the I-CAH Registry in November 2022 (https://sdmregistries.org/) and centers with eligible cases were invited to participate. All patients who were older than 18 years at the end of July 2022 or diagnosed with other forms of CAH were excluded. The I-CAH Registry is an international database of pseudonymized information on patients with CAH and is approved by the National Research Ethics Service in the United Kingdom as a research database of information that is collected as part of routine clinical care [17]. The data within the registry are deposited by clinicians following informed consent from patients or guardians. For the purpose of this study, the phenotypic classification into salt-wasting and simple virilizing CAH was based on concurrent treatment with fludrocortisone (FC).

### Clinical Data Collection

Clinicians with eligible cases were instructed to enter data on acute AI-related AEs for a minimum of 2 clinic visits per year per case for the period beginning August 2019 to the end of July 2022 . For consistency with the previous analysis performed in 2019, data from cases with even a single clinic visit were included in the analysis. SDEs and AC were based on the clinical judgment of the reporting clinician and were all associated with an increase in glucocorticoid (GC) dosing. Details of the data that were collected on these events have been previously described [6]. In cases where no visits had any data on acute AI-related AEs, the center was asked to confirm that the case had no events. Data were also gathered about GC and FC regimens at the time of clinic visits and which, as previously described, were based on individual center's practice [14]. These doses were converted to total hydrocortisone-equivalent GC dose as previously described [18]. GC and FC doses were categorized as low, normal, or high, based on Endocrine Society clinical practice guidance for 21-hydroxylase deficiency CAH as follows: GC low <10 mg/m^2^/day, normal 10 to 15 mg/m^2^/day, high >15 mg/m^2^/day; FC low <50 µg/day, normal 50-200 µg/day, and high >200 µg/day [19]. CAH-related hospitalization included attendance at emergency room, hospital admission, and intensive care unit admission in both exercises, as recorded in the longitudinal module in I-CAH. Cases that were recorded to be on FC were categorized as having salt-wasting CAH.

### Statistical Analysis

The observed frequency of SDE was determined as incidence rate, calculated as the number of SDE divided by person-years. For assessment of geographical differences in the occurrence of SDE, participating countries were categorized as those from a low- or-middle-income country or from a high-income country as defined by the 2023 World Bank classification [20]. Inter-group comparison for these variables was performed by the Mann-Whitney *U* test. The Fisher exact test was performed to compare proportions in different groups. Results were reported as frequencies, percentages, medians, and ranges. The centers that participated in the previous 2019 exercise had an intra-center comparison that enabled an assessment of change of each center's benchmark as well as the change of the overall I-CAH benchmark. SDE rates for 2019 and 2022 were displayed as funnel plots that were created using RStudio (v.12.0, Boston, MA, USA). Because the cohort studied in this study was restricted to the 3-year period of 2019 through 2022 and the previous cohort in 2019 did not have a restriction and included all data available up until that year, a further comparison was performed of the 2022 cohort with a cohort that just included data between 2016 to 2019. Further analyses that adjusted for age and phenotype (only salt-wasting [SW]) was also performed. All data analysis was performed using SPSS (v.25.0, Armonk, NY: IBM Corp) and GraphPad (v.10.1, Boston, MA, USA).

## Results

### Description of Cases and Comparison to the 2019 Cohort

A total of 942 patients from 48 centers were eligible for inclusion in the study. Data on AEs were available for 513 children and, of those, 257 (50%) were boys, 406 (79%) had SW CAH and 100 (20%) had simple virilizing CAH (Table 1). These children were reported from 38 centers in 21 countries with a median of 8 cases per center (range, 1-58). Within the 38 centers, there were 27 high-income country and 11 low- or-middle-income country centers. A total of 2470 clinic visits, occurring between August 2019 and August 2022, were evaluated in these 513 children, comprising a total of 880 patient-years (Table 1). The median duration of follow-up per patient was 2 years (0.1-2.9), with a median of 2.4 visits per patient-year (0.7-20.7). In the 2019 cohort, the median duration of follow-up per case was 3 years (0,1-17.9) with a median of 2.9 visits per patient-year (0.3-25.7). The median patient age at the time of each visit was 8.1 years (0-17.9) (Table 1). Of the 2172 visits where the GC dose was recorded, in 1288 visits (59%), the dose was within the recommended range, whereas in 439 visits (20%) this was high and in 445 visits (21%) low (Table 1). Of the 2470 clinic visits, hydrocortisone was the GC used in 2178 (88%), prednisolone in 18 (0.7%), dexamethasone in 15 (0.6%), and other or not reported in 226 (9.7%).

**Table 1.** Clinical variables of the 3 cohorts: (1) current cohort covering all visit data between 2019 and 2022, (2) previous cohort covering all visit data up to 2019 (1984-2019), and (3) all visit data between 2016 and 2019

	2019-2022	Prior to 2019	2016-2019
Centers (n)	38	32	27
Countries (n)	21	18	17
Patients (n)	513	516	222
Patients per age band at time of visit (n)			
<1 y	73	418	43
1-4.9 y	136	416	86
5-14.9 y	321	182	102
15-17.9 y	72	57	29
Visits (n)	2470	5388	786
Total patient-years	880	2300	256
Median number of children per center (range)	8 (1-58)	11 (1-53)	5 (1-27)
Median age, y (10th-90th centile)	8.1 (1.1-14.9)	2 (0.2-10.3)*^a^*	4.84 (0.2-14.6)^a^
M:F ratio (%)	50:50	47:53	45:55
LMIC:HIC ratio (%)	29:71	34:66	33: 67
CAH phenotype SW:SV:NK (%)	79:20:1	89:11:0*^a^*	92:8%:0*^a^*
Number of visits with GC dose data (%)	2172 (88%)	4226 (78%)	551 (70%)
Daily GC dose Normal:high:low (%)	59:20:21	48:28:24*^a^*	49:22:29*^a^*
Median SDE per patient-y per center (range)	0 (0-2.5)	0.3 (0-6)*^a^*	0.55 (0-11.2)*^a^*
Median AC per patient-y per center (range)	0 (0-5.3)	0 (0-3)	0 (0-1.4)
Median hospitalization per patient-y per center (range)	0 (0-1)	0 (0-2.2)	0 (0-6.9)

Abbreviations: AC, adrenal crisis; CAH, congenital adrenal hyperplasia; GC, glucocorticoid; HIC, high income countries; LMIC, low- to middle-income country; NK, not known; SDE, sick day episode; SV, simple virilizing; SW, salt wasting.

The sum of the patients in each age band exceeds the total number of patients as the age refers to the decimal age at the time of visit and some patients had visits in more than 1 age band.

^*a*^ *P* < .01, *^a^P* = .01.

### Trends in Acute AI-related AE Rates

**Figure 1.**
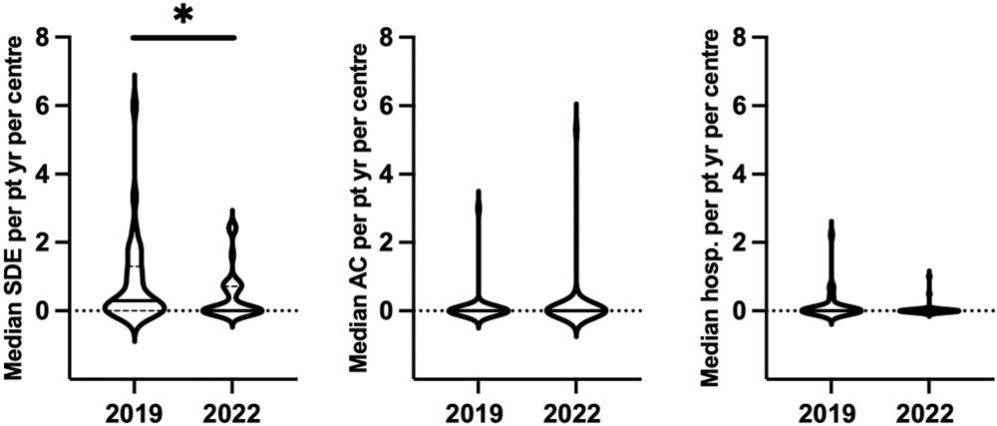
Violin plots showing the median adrenal insufficiency-related adverse events rate sick day episode (SDE), adrenal crises (AC) and hospitalization per patient-year in the 2019 cohort (covering visit data between 1984 and 2019) and the 2022 cohort (covering visit data between 2019 and 2022). *P = .01.

**Figure 2.**
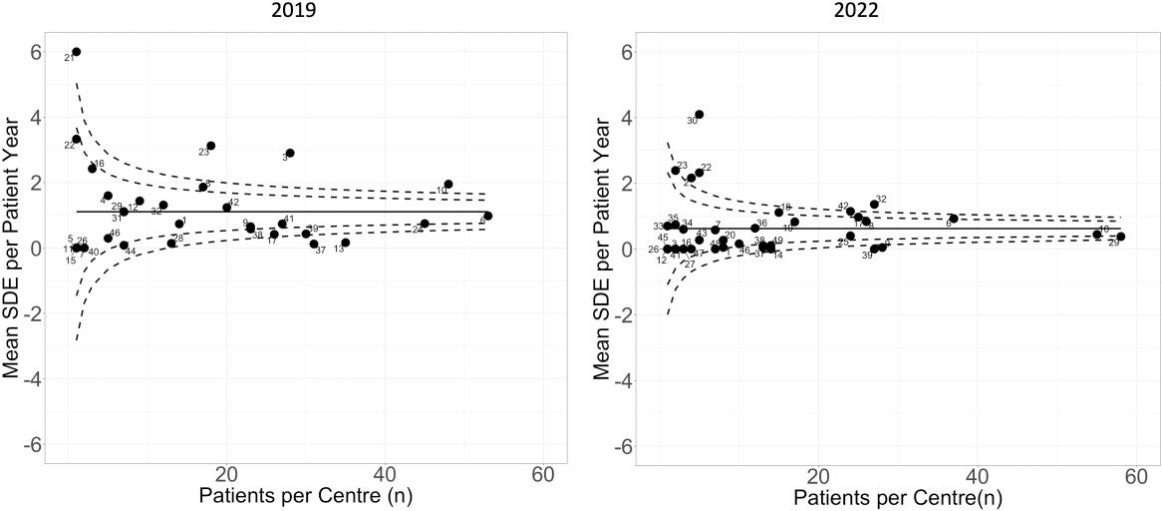
Funnel plots showing the sick day episode (SDE) benchmarks in 2019 and 2022. The continuous line indicates the overall mean SDE for the 2019 and 2022 cohort. Each center is represented by a number, which is the same if they participated in the 2019 and 2022 cohorts. The dotted lines indicate the 2 SD and 3 SD from the overall mean.

**Figure 3.**
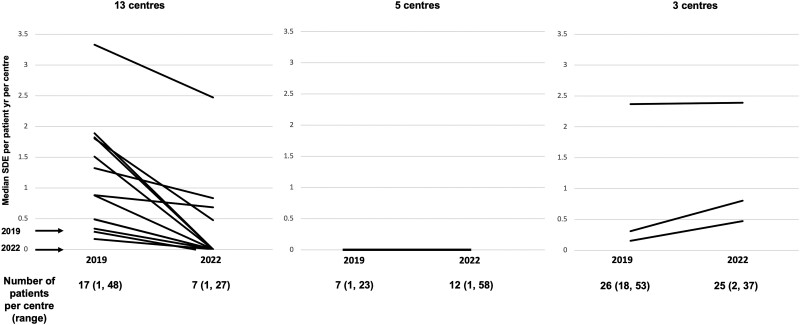
Change in reported sick day episode (SDE) rates in the 21 centers that participated in both 2019 and 2022 cohorts expressed as median SDE per patient-year per center. Reduction in SDE per patient year was noted in 13 centers, in 5 centers the SDE rate remained stable, and increased SDE rate was noted in 3 centers.

**Figure 4.**
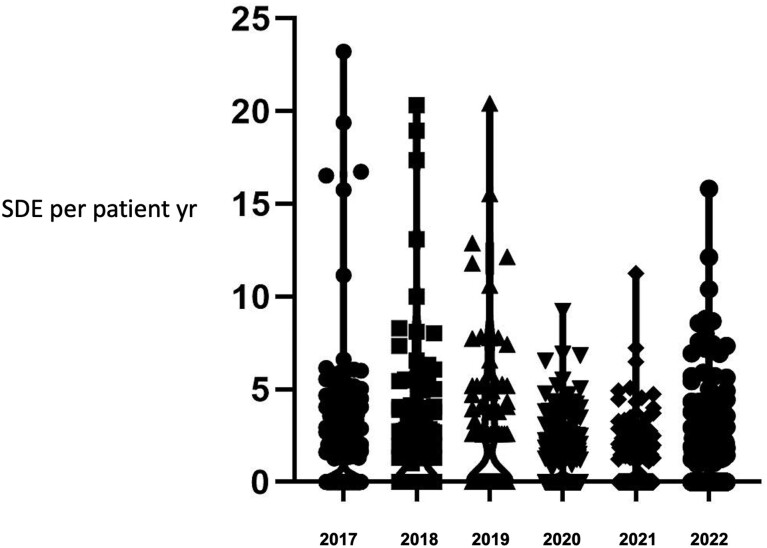
Trend of SDEs per patient year in yearly intervals during 2016-2022.

The median SDE per patient-year per center was 0 (0-2.5) in 2022 compared to 0.3 (0-6) in 2019 (*P* = .01) (Fig. 1). The data for each center that participated in 2019 and in 2022 showed a reduction in SDE rate across all centers (Fig. 2). Although the reported SDE rate were much more variable in the centers that had included a smaller number of cases (Fig. 2), the reduction in SDE rate was generally observed in most centers regardless of the case load (Fig. 3). Of the 38 centers in the 2022 cohort, 21 (55%) had also participated in 2019 and a reduction in SDE per patient-year was noted in 13 (62%) centers, whereas in 5 (24%) the SDE rate remained the same and in 3 (14%) it showed an increase (Fig. 3). Of the 474 SDEs reported in the 2022 cohort, infectious illnesses were reported as the precipitating factor in 287 (61%) compared to 1105 of 1544 SDE events in the 2019 cohort (72%) (*P* < .001). Other precipitating factors for the 474 SDEs in the 2022 cohort included surgery in 12 (3%) and miscellaneous in 43 (9%). The median SDE per patient-year was also higher in the 2016 through 2019 cohort at 0.55 (0-11.2) compared to 0 (0-2.5) in the 2019 to 2022 cohort (*P* = .007). The median hospitalization rate per patient-year per center was not different between the 2 cohorts, at 0 (0-1) in 2022 compared to 0 (0-2.2) in 2019 (*P* = .06) (Fig. 1). This was also similar to the rate of the 2016 to 2019 cohort (*P* = .9). Of the 474 SDEs reported in 2022, 103 (22%) led to hospitalization and this proportion was lower than that reported in 2019 when 299 SDEs of a total of 1099 (27%) led to hospitalization (*P* = .02). When compared to the 2016 to 2019 data, the hospitalization rate, at 17% (66 SDEs of 296 SDEs reported in total), was not statistically significantly different compared to 2022, *P* = .06. The median rate of AC per patient-year per center was 0 (0-5.3) in 2022 and 0 (0-3) in 2019 (*P* = .5) (Fig. 1). This did not differ significantly compared to the 2016 to 2019 cohort either (*P* > .05). The trend of SDEs per patient year in yearly intervals during 2016 to 2022 is shown in Fig. 4.

### SDE Rate Analysis Based on CAH Phenotype and Age Categories

**Figure 5.**
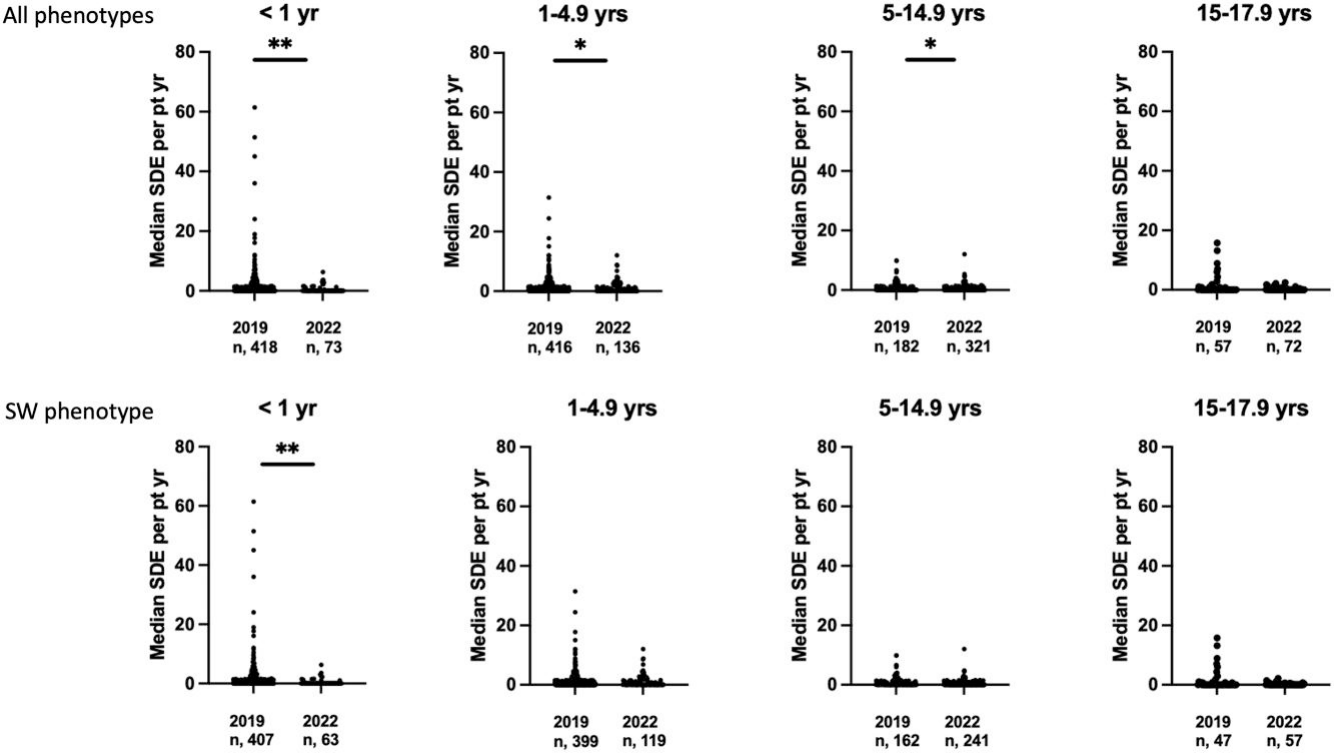
Median sick day episode (SDE) per patient-year by age bands for all phenotypes and for only the salt-wasting (SW) phenotype based on the data from the 2019 and 2022 cohorts, respectively. **P < .01 and *P < .05.

Because the age of the children in the 2022 cohort was older and the proportion of children with SW CAH was lower (Table 1), the analysis was also performed after adjustment for these variables. The relevant information is shown in Table 1. After adjustment for the SW phenotype, the median SDE per patient-year remained higher in 2019 compared to 2022 at 0.38 (0-13.3) vs 0 (0-12), *P* < .01 respectively. All age bands, except for the oldest one, were observed to have higher SDE rates in the 2019 cohort (Fig. 5). Similar analysis was performed following stratification for a combination of age categories at time of visit and phenotype. The statistically significant differences were eliminated except for those with a SW group who were younger than age 1 year (Fig. 5). In the 2019 to 2022 cohort, of the 513 patients, age of first presentation was available in 492 cases and, of these, 483 (98%) first presented before the age of 8 years (females:males, 233:249), whereas 9 (1.8%) had their first presentation between the ages of 8 and 12 years (female:males, 3:6) and only 1 female first presented between the age of 12 and 16 years.

## Discussion

We report the first multicenter, international study that has evaluated changes in the occurrence of acute AI-related AE in children with 21-OHD CAH. Although other studies have focused on ACs in adolescence and adulthood [11, 12], the current study has reported on multiple outcomes related to CAH, including the occurrence of SDEs, Acs, and hospitalization rates in children aged 0 to 18 years. The study also provides a framework for the continued surveillance of AI-related AEs in CAH that can support a long-term quality improvement exercise. We show that the period between 2019 and 2022 was associated with a reduction in reported SDE compared to the original report in 2019 [6] and there may be several reasons for this observation.

Previous analysis of the I-CAH cohort had reported that factors associated with a higher likelihood of SDE included an age between 1 and 4 years and 15 and 18 years and an SW phenotype, whereas a higher hydrocortisone dose had been associated with a lower likelihood of SDE [6]. The cohort in the current study was indeed older and had a lower proportion of children with SW CAH but adjusting for these 2 variables did not change the observation of a reduction in SDE rates. Given that almost all the cases in the 2022 cohort had presented in early childhood, the likelihood of any of them having nonclassical forms of 21-OHD CAH was also highly unlikely. It is also notable that the 2022 cohort had a lower proportion of cases on a higher dose of hydrocortisone and despite this, the cohort displayed a lower SDE. We therefore believe that case selection was not a plausible explanation for the observed fall in SDE rate. In the cohorts studies, only 10% to 20% of the cohorts were classed as not having SW CAH based on the fact that they were not reported to be on FC at the visit. However, it is possible that these cases, labelled as simple virilizing may have still had SW given that this condition may be a continuum in CAH [1]. Furthermore, drug availability may vary among different countries and a limited availability of FC could also play a role in the occurrence of SDE.

Given that the definition of SDE is not robust and may be prone to variation between reporting centers [9], it is possible that there may have been interobserver reporter variation or new centers that joined the exercise after 2019 had a different definition of SDE. However, the reduction in SDE was observed in most of the centers that participated in both 2019 and 2022. Adrenal crises in childhood CAH are rarely observed [6] and it is therefore not surprising that no clear change was observed for this event.

Viral illnesses are a common contributory factor for SDE in children [21] and it is possible that an explanation for the observed decrease in SDEs was the reduced incidence and transmission of pediatric viral and bacterial illnesses because of the lock-down during the COVID-19 pandemic period [22-25], which coincided with the 3-year period up to 2022. Although it is possible that inadequately controlled cases of primary adrenal insufficiency because of CAH were at an increased risk of acute AI-related AEs during the pandemic [26-28], the current study agrees with other observations that children with CAH were not at an increased risk of severe AI-related AEs during the pandemic period [29]. It is also possible that the health care strains during the pandemic may have altered the clinical encounters between patients, parents, and health care providers and affected recall or reporting of SDE. The recall bias could also have been introduced by health care professionals who may not have reported the SDEs as assiduously as before. The observation of a fall in the proportion of SDE cases that led to a hospitalization may also have been related to the pandemic, although it is also possible that this may have been due to better management of the SDE at home. Another possible bias may have been introduced because of some centers recruiting a limited proportion of cases at their center. However, it was reassuring to see that the funnel plot analysis showed that a reduction in SDE was observed regardless of the number of cases at any center. Nevertheless, studying these trends for longer will allow exploration of these possible explanations in the future.

Following the initial benchmarking exercise in 2019, participating centers had each received a center-specific report of their center's acute AI-related AE rate in comparison to the overall benchmark for all participating centers. Benchmarking in clinical practice can potentially contribute to improving the quality of care in health care settings through a variety of methods including identification of best practices, performance measurement, resource allocation, greater focus on patient outcomes, standardization of processes, and informed decision making [30, 31]. It is possible that the feedback that was provided to the centers through the benchmarking reports in 2019 acted as a tool that raised awareness of acute adrenal insufficiency-related adverse events among participating centers and promoted care quality improvement. Strategies to reduce adrenal crises in adrenal insufficiency, including structured and repeated patient education, have been previously explored [32-34]. It is possible that the different centers may have employed different strategies, and this needs further investigation to understand practices that may have contributed to the observed improvement. As the I-CAH dataset continues to increase and enrich with more cases, it is possible that in the future there may be a role for exploring a relationship of acute AI-related AE to the specific genotype.

In summary, the I-CAH Registry can be used for targeted monitoring of important clinical benchmarks. However, interpretation of data needs to be undertaken carefully. Among participating centers, a clear reduction of SDEs was observed between 2019 and 2022 in children with CAH. There is a need to explore the factors that may have influenced this change and this will be achieved as the exercise is repeated again over the longer term.

## Data Availability

Some or all datasets generated during and/or analyzed during the current study are not publicly available but are available from the corresponding author on reasonable request.

## Abbreviations

21-OHD: 21-hydroxylase deficiency
AC: adrenal crisis
AE: adverse event
AI: adrenal insufficiency
CAH: congenital adrenal hyperplasia
FC: fludrocortisone
GC: glucocorticoid
SDE: sick day episode
SW: salt wasting
